# Lipopolysaccharide-induced neuroinflammation leads to the accumulation of ubiquitinated proteins and increases susceptibility to neurodegeneration induced by proteasome inhibition in rat hippocampus

**DOI:** 10.1186/1742-2094-9-87

**Published:** 2012-05-04

**Authors:** Cristina Pintado, María P Gavilán, Elena Gavilán, Luisa García-Cuervo, Antonia Gutiérrez, Javier Vitorica, Angélica Castaño, Rosa M Ríos, Diego Ruano

**Affiliations:** 1 Departamento de Bioquímica y Biología Molecular, Facultad de Farmacia, Universidad de Sevilla, 41012, Sevilla, Spain; 2 Centro Andaluz de Biología y Medicina Regenerativa (CABIMER), 41092, Sevilla, Spain; 3 Instituto de Biomedicina de Sevilla (IBIS)-Hospital, Universitario Virgen del Rocío/Consejo Superior de Investigaciones Científicas/Universidad de Sevilla, 41013, Sevilla, Spain; 4 Centro de Investigación Biomédica en Red sobre Enfermedades Neurodegenerativas (CIBERNED), 41013, Sevilla, Spain; 5 Departamento de Biología Celular, Genética y Fisiología, Facultad de Ciencias, Universidad de Málaga, Málaga, Spain

**Keywords:** Immunoproteasome, Neurodegeneration, Neurodegenerative diseases, Neuroinflammation, Protein accumulation

## Abstract

****Background**:**

Neuroinflammation and protein accumulation are characteristic hallmarks of both normal aging and age-related neurodegenerative diseases. However, the relationship between these factors in neurodegenerative processes is poorly understood. We have previously shown that proteasome inhibition produced higher neurodegeneration in aged than in young rats, suggesting that other additional age-related events could be involved in neurodegeneration. We evaluated the role of lipopolysaccharide (LPS)-induced neuroinflammation as a potential synergic risk factor for hippocampal neurodegeneration induced by proteasome inhibition.

****Methods**:**

Young male Wistar rats were injected with 1 μL of saline or LPS (5 mg/mL) into the hippocampus to evaluate the effect of LPS-induced neuroinflammation on protein homeostasis. The synergic effect of LPS and proteasome inhibition was analyzed in young rats that first received 1 μL of LPS and 24 h later 1 μL (5 mg/mL) of the proteasome inhibitor lactacystin. Animals were sacrificed at different times post-injection and hippocampi isolated and processed for gene expression analysis by real-time polymerase chain reaction; protein expression analysis by western blots; proteasome activity by fluorescence spectroscopy; immunofluorescence analysis by confocal microscopy; and degeneration assay by Fluoro-Jade B staining.

****Results**:**

LPS injection produced the accumulation of ubiquitinated proteins in hippocampal neurons, increased expression of the E2 ubiquitin-conjugating enzyme UB2L6, decreased proteasome activity and increased immunoproteasome content. However, LPS injection was not sufficient to produce neurodegeneration. The combination of neuroinflammation and proteasome inhibition leads to higher neuronal accumulation of ubiquitinated proteins, predominant expression of pro-apoptotic markers and increased neurodegeneration, when compared with LPS or lactacystin (LT) injection alone.

****Conclusions**:**

Our results identify neuroinflammation as a risk factor that increases susceptibility to neurodegeneration induced by proteasome inhibition. These results highlight the modulation of neuroinflammation as a mechanism for neuronal protection that could be relevant in situations where both factors are present, such as aging and neurodegenerative diseases.

## Background

Neuroinflammation has distinct features that are shared in aging and in neurodegenerative diseases. Microglia are the main immune cell in the brain, playing a role in both physiological and pathological conditions [1-5]. Although acute neuroinflammation plays a protective role [6-8], chronic neuroinflammation is frequently considered detrimental and damaging to nervous tissue [2,3]. Thus, whether neuroinflammation has beneficial or harmful outcomes in the brain may critically depend on both the duration of the inflammatory response and the kind of microglial activation [9]. As the primary source for proinflammatory cytokines, microglia are implicated as a pivotal mediator of neuroinflammation and can induce or modulate a broad spectrum of cellular responses [10].

In relation to protein homeostasis, some proinflammatory cytokines, such as IFN-γ and TNF-α, can alter the proteolytic activity of the proteasome, leading to the switch to immunoproteasome (i-proteasome) [11,12]. The proteasome is a molecular complex that controls intracellular protein homeostasis by degrading misfolded and/or regulatory proteins. It is made up of the 20 S-proteasome, a central unit carrying the catalytic activities, and several regulatory complexes such as PA700/19 S or PA28/11 S [13]. The 26 S-proteasome (19 S-20 S-19 S) is responsible for the catalysis of the ATP-dependent degradation of polyubiquitinated proteins formed by a cascade of E1, E2 and E3 enzymes, which activate, conjugate and transfer, respectively, multiple ubiquitin molecules to protein substrates, thus targeting these for degradation [13-15].

As mentioned before, following IFN-γ or TNF-α stimulation, or after LPS injection, the constitutive catalytic subunits β1, β2 and β5 are replaced by the inducible catalytic subunits β1i, β2i and β5i, in order to form the i-proteasome that associates with the regulatory complex PA28/11 S [12,16,17]. Because substrates of proteasomes are short-lived regulatory proteins involved in cell differentiation, cell-cycle regulation, transcriptional regulation or apoptosis [18], a rapid and efficient elimination of proteins by the ubiquitin proteasome system (UPS) is essential under stress conditions that cause the accumulation of misfolded or partially denatured proteins [19].

Despite neuroinflammation and proteasome dysfunction being two significant hallmarks in many neurodegenerative diseases, the relationship between the factors is poorly explored. Here, we have evaluated the potential role of neuroinflammation on the UPS. Our results provide strong evidence supporting a synergic effect of neuroinflammation and proteasome dysfunction for hippocampal neurodegeneration.

## Material and methods

### **Animals**

Young (3 to 4 months) and aged (24 to 26 months) male Wistar rats were provided by the animal care facility of the University of Seville. All experiments were approved by local ethical committees and complied with international animal welfare guidelines.

### **Surgery**

Young male Wistar rats (200 to 250 g; n = 58) were processed for surgery as previously described [20,21]. Different groups of animals were established, rats injected with LPS, rats injected with saline + LPS or saline + LT or LPS + LT, and control rats injected with saline only.

For the rats injected with LPS, the LPS (Sigma-Aldrich, St Louis, MO, USA) was dissolved (5 mg/mL) in a solution of sterilized PBS and 1 μL was injected into both hippocampi. The rats were anesthetized with 400 mg/kg chloral hydrate and positioned in a stereotaxic apparatus (Kopf Instruments, Tujunga, CA, USA). According to Paxinos’ atlas, the coordinates were: 3.3 mm posterior, 1.6 mm lateral and 3.2 mm ventral to the bregma and 4.8 mm posterior, 5.5 mm lateral and 6.0 mm ventral to the bregma. The injections were delivered over a period of 2 min and the needle was left *in situ* for an additional 5 min to avoid reflux along the injection track. Animals were decapitated at 3 hours, 6 hours, 14 hours, 24 hours, 3 days and 7 days after LPS injection and brains were quickly removed. Control animals were processed similarly but received 1 μL of sterilized PBS in both hippocampi.

The procedure for rats injected with saline + LPS or saline + LT or LPS + LT, the LT (Sigma-Aldrich) was dissolved (5 mg/mL) in a solution of sterilized PBS and 1 μL was injected into both hippocampi. For each case, saline or LPS was first administered and 24 hours later, LPS or LT was injected through the same drilled hole. Finally, animals were sacrificed 48 hours after the last injection. In addition, male Wistar aged rats (24-month-old, n = 3) were included in the saline + LT-injected group. Animals were processed similarly but the coordinates were 6.0 mm posterior, ± 4.6 mm lateral and 4.6 mm ventral to the bregma as previously shown [22].

### **Sample preparation**

Both hippocampi were dissected, frozen in liquid N_2_ and stored at −80°C until use. Hippocampi were homogenized in 700 μL of ice cold sucrose buffer (0.25 M sucrose, 1 mM ethylenediaminetetraacetic acid, 10 mM Tris–HCl, pH 7.4) supplemented with a protease inhibitor cocktail (Sigma-Aldrich). Three hundred microliters were separated and used for RNA isolation (see below). The remaining homogenized solution (400 μL) was centrifuged at 15,000 × g for 30 min at 4°C and the supernatant was recovered and stored at −80°C until use. Protein concentration was determined by the Lowry method.

### **RNA extraction, reverse transcription and real-time PCR**

Total RNA extraction and reverse transcription was carried out with 300 μL of each homogenized hippocampi sample as previously described [22]. Real-time PCR was performed in an ABI Prism 7000 sequence detector (Applied Biosystems, Madrid, Spain) using cDNA diluted in sterile water as a template. Analyzed genes were amplified using specific Taqman probes supplied by Applied Biosystems. Threshold cycle (Ct) values were calculated using the software supplied by Applied Biosystems.

### **Proteasome activity assay**

Proteasome activity was determined in hippocampal samples using specific fluorogenic substrates for the chymotrypsin activity of the proteasome. Proteasome activity was abolished in the presence of 10 μM MG-132 [22].

### **Antibodies and immunoblots**

The following primary antibodies were used in this study. Rabbit polyclonal anti-inducible nitric oxide synthase (iNOS; BD Bioscience, San José, CA, USA), anti-ubiquitin (Dako, Glostrup, Denmark); anti-β5i subunit (Abcam, Cambridge, UK), anti-proteasome maturation protein (POMP; Biomol, Madrid, Spain), anti-Bax, anti-Bak, anti-B-cell lymphoma extra large (Bcl-XL) and anti-Bcl-2 (Cell Signaling, Danvers, MA, USA), and anti-caspase-3 (Stressgen, Ann Arbor, MI, USA); mouse monoclonal anti-β-actin (Sigma-Aldrich) and anti-neuronal nuclei (NeuN; Chemicon, Billerica, MD, USA); horseradish-peroxidase-conjugated corresponding secondary antibodies (Dako); and secondary antibodies conjugated to DyLight fluorophores (Jackson Inmunoresearch, Madrid, Spain). Immunoblots were performed as previously described [20,22].

### **Immunofluorescence and confocal microscopy**

Animals were transcardially perfused with 4% paraformaldehyde and brains were processed as previously described [2]. Sections 25 μm-thick were cut on a cryostat and mounted on gelatin-coated slides, permeabilized with 0.5% Triton (Sigma-Aldrich) overnight at room temperature, incubated with primary antibody anti-ubiquitin for 1 h at room temperature and overnight at 4°C and, finally, with the appropriate DyLight^TM^-conjugated-secondary antibodies for 1 h. Nuclei were counterstained with 4′-6-diamidino-2-phenylindole (DAPI) at a final concentration of 1 ng/μL after secondary antibody labeling. Control staining included omission of primary antibodies or irrelevant primary antibodies of the same isotype. Then, sections were washed and coverslipped with 0.01 M PBS containing 50% glycerin and 2.5% triethylenediamine and examined under a motorized upright wide-field microscope (Leica DM6000B). Confocal images were captured using a TCS SP5 Confocal Leica laser scanning microscope equipped with a DMI60000 microscope and an HCX PL APO lambda blue 63× 1.4 oil objective at 22°C. Maximum projection image was obtained.

### **Statistical analysis**

Statistical analysis was performed using the Statgraphics plus (v 3.1) software. The differences between groups in the time-course experiments were assessed by one-way analysis of variance followed by Turkey’s test. The data comparison between the saline and saline + LT, saline + LPS and LPS + LT animals was carried out using two-tailed *t*-test. The significance was set at *P* <0.05. Significant differences are indicated by an asterisk.

## Results

### **LPS injection increases the content of ubiquitinated proteins in hippocampal neurons**

**Figure 1:**
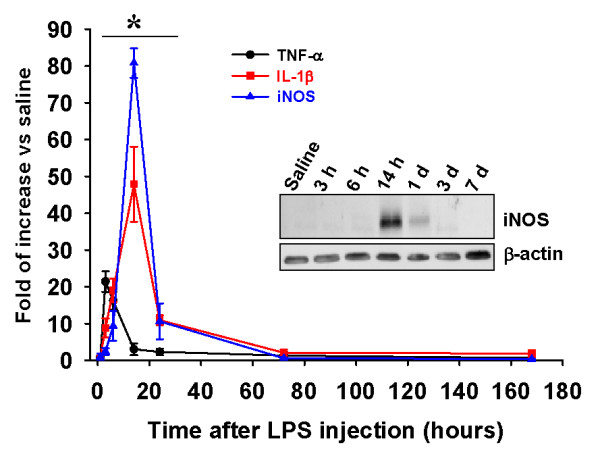
**Temporal changes in the expression of TNF-α, IL1-β and iNOS mRNAs in the rat hippocampus after LPS injection.** The expression of mRNA coding for the proinflammatory cytokines TNF-α and IL1-β and for the enzyme iNOS was quantified at different times post-injection (from 3 hours to 7 days) in samples from hippocampi injected with 5 μg of LPS. Data are presented as the mean ± SD relative to saline-injected animals. Data were obtained from triplicate assays. In the insert is shown a representative western blot for the iNOS enzyme in samples from the same animals used for gene expression. *P <0.05. iNOS: inducible nitric oxide synthase; IL1- β: interleukin-1 beta; LPS: lipopolysaccharide; SD: standard deviation; TNF-α: tumor necrosis factor alpha.

**Figure 2:**
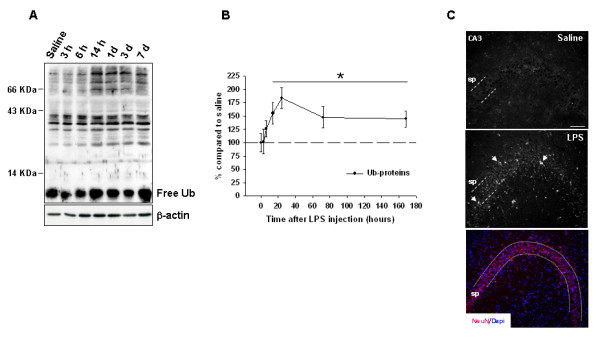
**Accumulation of ubiquitinated proteins induced by LPS injection. (A)** Ubiquitinated proteins, predominantly those of high molecular weight, accumulated in young rat hippocampi after LPS injection. Shown is a representative western blot experiment corresponding to saline- and LPS-injected animals sacrificed at indicated times. Each line corresponds to four animals pooled and the experiment was repeated at least three times with similar results. **(B)** Densitometric quantification of western blot experiments (n = 3). The full line, without the inclusion of the free ubiquitin, was considered for the analysis. **(C)** Cellular distribution of ubiquitinated proteins in the CA3 region of saline (upper) and 3 days after LPS injection (middle). Most of the immunostaining was observed in the stratum pyramidale, where the somata of principal neurons are located as revealed by the intense immunostaining of the neuronal marker NeuN (lower). Nuclei were counterstained with DAPI. *P <0.05 significant difference with respect to saline-injected animals. DAPI: 4′-6-diamidino-2-phenylindole; LPS: lipopolysaccharide; NeuN: neuronal nuclei; sp: stratum pyramidale. Scale bar: 75 μm.

To analyze the role of LPS-induced neuroinflammation on the UPS, we induced neuroinflammation in young rat hippocampi by intrahippocampal injection of LPS. As expected [21], LPS injection induced a significant increase (*P* <0.05) early on in the mRNA expression of proinflammatory factors such cytokines TNF-α and IL-1β, and the enzyme iNOS (Figure 1), indicating the induction of neuroinflammation. Interestingly, LPS injection also increased the content of ubiquitinated proteins, mostly those with high molecular weight (Figure 2A). The content of these ubiquitinated proteins was significantly higher than in saline-injected rats from 14 hours to even 7 days after LPS injection (*P* <0.05; Figure 2B). Immunofluorescence experiments revealed that ubiquitinated proteins accumulated preferentially in the stratum pyramidale of the CA3 (Figure 2C) and CA1 regions (data not shown), where the cell bodies of principal neurons localize as revealed by NeuN immunostaining (Figure 2C lower panel).

### **LPS injection up-regulates the mRNA expression of the E2 ubiquitin-conjugating enzyme UB2L6, decreases proteasome activity and increases immunoproteasome biogenesis**

**Figure 3:**
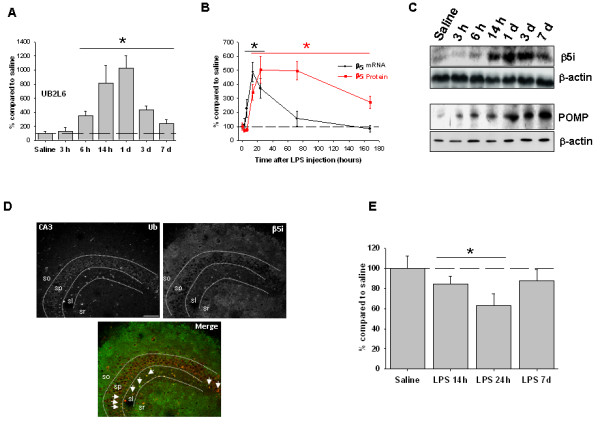
**Modification of the UPS induced by intrahippocampal LPS injection. (A)** Profile of the mRNA expression of the E2 ubiquitin-conjugating enzyme UB2L6 at different times post-LPS injection. Data are presented as the mean ± SD relative to saline-injected animals. Data were obtained from triplicate assays. **(B)** Profile of the mRNA expression of the β5i subunit (black line) in the same animals as in A. **(C)** At the top is shown a representative western blot of the β5i subunit (mature form) in samples from rats injected with saline and LPS, sacrificed at times indicated. Densitometric quantification of western blot experiments (n = 3) is shown in B (red line). At the bottom is shown a representative western blot of POMP in the same animals as shown before. The experiment was repeated three times with similar results. Note the strong time-dependent increase in the content of this protein involved in proteasome assembly, indicating an active process of *de novo* proteasome biogenesis. **(D)** Immunofluorescence analysis of the cellular distribution of ubiquitinated proteins and the β5i in hippocampal slices from LPS-injected rats. Images correspond to animals sacrificed 3 days after LPS injection. The β5i immunostaining was mainly located in neuronal fibers in the stratum oriens and stratum radiatum. Some scattered cellular bodies were also observed in the stratum lucidum. Arrows indicate colocalization of ubiquitin and β5i immunostaining. Bar scale 75 μm. **(E)** Chymotrypsin-like activity was analyzed in saline- and LPS-injected rats at different times post-injection (n = 4 per time point). LPS injection significantly decreased the chymotrypsin activity of hippocampal proteasomes. *P <0.05. LPS: lipopolysaccharide; POMP: proteasome maturation protein; sl: stratum lucidum; so: stratum oriens; sp: stratum pyramidale; sr: stratum radiatum.

We next investigated for potential factors that could explain the LPS-induced accumulation of ubiquitinated proteins. In this sense, and based on data by others [23], we considered the possibility that ubiquitin conjugation activity increases after LPS injection. So, we analyzed the mRNA expression of the E2 ubiquitin-conjugating enzyme UB2L6 at different times post-LPS injection. As shown in Figure 3A, the mRNA expression of the E2 UB2L6 enzyme was transcriptionally up-regulated in a time-dependent manner after LPS injection, being significantly increased from 6 hours to 7 days after LPS injection (*P* <0.05). In addition, LPS injection produced an early and concomitant transcriptional up-regulation of the rate-limiting i-proteasome subunit β5i, followed by protein synthesis and i-proteasome biogenesis, as reflected by the increase in both the mature form of the β5i subunit [21] and POMP [24,25] (Figure 3B, C). The β5i subunit was diffusely expressed in the strata oriens and radiatum of the LPS-injected rats (72 hours after LPS injection), suggesting a preferential expression in neuronal projections rather than in neuronal bodies. Some cellular bodies scattered in the stratum lucidum of the hippocampus were also observed. These could correspond to interneurons or glial cells. Importantly, some degree of co-distribution between ubiquitin and β5i subunit was observed in some pyramidal cells and other cells in the stratum lucidum (Figure 3D). The LPS injection also resulted in a transient decrease of proteasomal chymotrypsin activity 14 to 24 hours after injection, which was restored 7 days later (Figure 3E), probably as consequence of the shift from constitutive to i-proteasome. Taken together, these data indicated that LPS injection transitorily alters protein homeostasis in hippocampal neurons. Importantly, this homeostatic alteration seemed to be not deleterious for neurons because no evident signs of neurodegeneration were observed (see below).

### **LPS-induced neuroinflammation increases neurodegeneration produced by proteasome inhibition**

Because LPS injection increased the content of ubiquitinated proteins in neurons and decreased proteasome activity, we wondered whether neuroinflammation could increase susceptibility to cellular death induced by proteasome inhibition. For that, we produced proteasome inhibition 24 h after LPS injection, exactly when ubiquitinated proteins accumulated and proteasome activity was decreased (see above).

**Figure 4:**
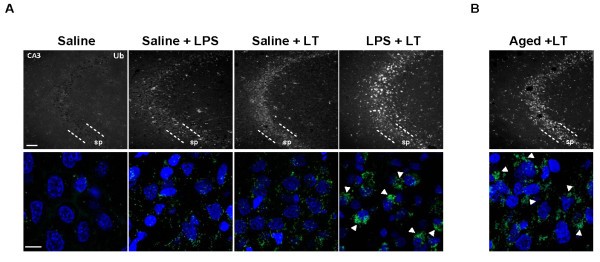
**Proteasome inhibition after intrahippocampal injection of LPS induced the formation of aggresome-like structures in hippocampal neurons. (A)** Upper: immunofluorescence analysis of the cellular distribution of ubiquitinated proteins in hippocampal slices from rats treated with saline, saline + LPS, saline + LT and LPS + LT. Images correspond to animals sacrificed 48 hours after the last injection. Lower: higher magnification corresponding to neurons from the stratum pyramidale taken from images showed above. Nuclei were counterstained with DAPI. Note the presence of large aggresome-like structures (arrowheads) only in the LPS + LT-injected rats. Bar scale: 75 μm for upper images and 10 μm for lower images. **(B)** Similar as in A but images correspond to aged rat hippocampus injected with LT alone. DAPI: 4′-6-diamidino-2-phenylindole; LPS: lipopolysaccharide; LT: lactacystin; sp: stratum pyramidale.

First, we analyzed at cellular level the distribution of ubiquitinated proteins induced by saline + LPS, saline + LT or LPS + LT injection at 72 hours (see Material and methods). As shown in Figure 4A (upper panel), ubiquitinated proteins were mostly localized in pyramidal somata of the CA3 region regardless of the treatment. A similar distribution was observed for the CA1 region (data not shown). Importantly, in the LPS + LT group, the intensity of ubiquitin immunostaining was increased. Indeed, higher magnification images from pyramidal cells revealed that accumulation of ubiquitinated proteins in the CA3 neurons of the LPS + LT animals concentrated mostly in perinuclear regions as bigger structures. By contrast, in both saline + LPS- or saline + LT-treated animals, ubiquitinated proteins appeared distributed throughout the cytoplasm as small punctuated structures (Figure 4A, lower panel). To further test this potential synergism between neuroinflammation and protein accumulation in a more physiological situation, we blocked the proteasome in aged rat hippocampus where neuroinflammation was widespread [2,4], and analyzed at cellular level the distribution of ubiquitinated proteins similarly as done in young rats. As show in Figure 4B, accumulation of ubiquitinated proteins was also observed in the CA3 region (and also in the CA1, data not shown) and, importantly, higher magnification images of pyramidal cells revealed accumulation of ubiquitinated proteins in perinuclear regions as bigger structures, similarly as observed in young LPS + LT-treated rats. Thus, these data strongly indicate that age-related chronic neuroinflammation is a synergic risk factor for protein accumulation.

**Figure 5:**
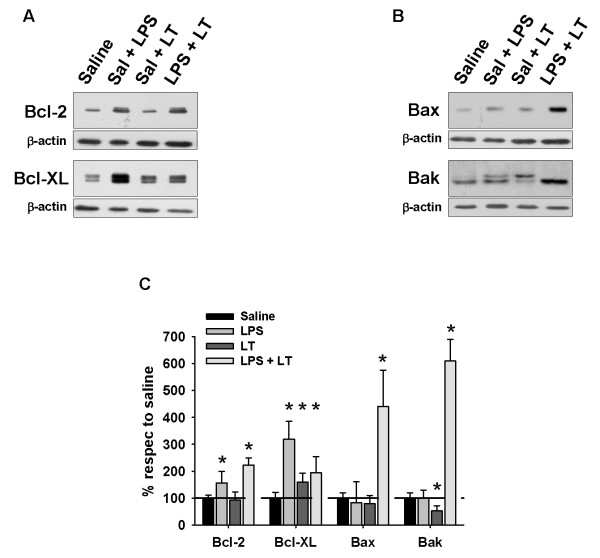
**Anti- and pro-apoptotic proteins expressed following saline, saline + LPS, saline + LT and LPS + LT injection in rat hippocampus. (A)** Representative western blots showing the expression of the anti-apoptotic proteins Bcl2 and Bcl-XL at 24 hours after saline + LPS, saline + LT or LPS + LT injection. **(B)** Similar as in A but for the pro-apoptotic proteins Bax and Bak. Experiment were done in parallel and repeated four times with similar results. **(C)** Densitometric analysis of western blots. Results were normalized with β-actin and referred to saline-injected animals. Data are presented as mean ± SD compared to a pool of saline-injected young animals. *P <0.05. Bcl: B-cell lymphoma; LPS: lipopolysaccharide; LT: lactacystin; SD: standard deviation.

**Figure 6:**
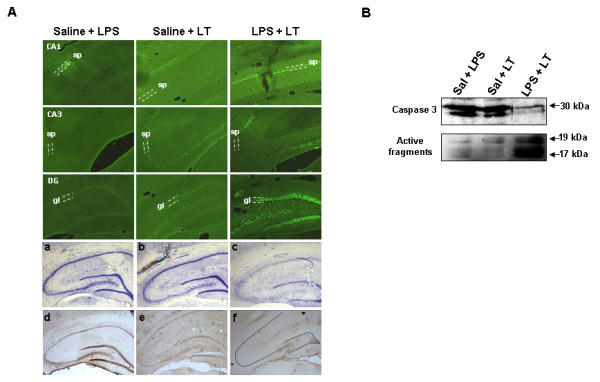
**LPS + LT injections increase hippocampal neurodegeneration in young animals. (A)** Fluoro-Jade B staining of brain slices of animals 72 hours after saline + LPS, saline + LT or LPS + LT injection. Images correspond to the CA1, CA3 and dentate gyrus (DG) of the hippocampal formation. Note the absence of neurodegeneration in saline + LPS rats, agreeing with biochemical data. Saline + LT injection produced a moderate neurodegeneration, but the number of positive Fluoro-Jade B cells was clearly increased in rats injected with LPS + LT. Representative photographs with cresyl violet staining are shown in the middle panel: (a) saline + LPS; (b) saline + LT; and (c) LPS + LT. In the lower panel is shown the immunohistochemistry detection of the neuronal marker NeuN in hippocampal slices from rats injected with (d)saline + LPS; (e) saline + LT and (f) LPS + LT. Note the strong diminution in the immunolabeling intensity for this neuronal marker in rats injected with LPS + LT. For orientative purposes, dotted lines represent the localization of the stratum pyramidale and granular layer, where somata of principal neurons are located. **(B)** Representative western blot of caspase-3. The amount of processed caspase-3 was higher in the LPS + LT rats in agreement with biochemical and cellular data. Image corresponding to the processed fragments is overexposed for a better visualization. Experiments were done in parallel and repeated at least three times with similar results. DG: dentate gyrus; gl: granular layer; LPS: lipopolysaccharide; LT: lactacystin; sp: stratum pyramidale.

To investigate whether differences in the manner of accumulation of ubiquitinated proteins could be related to neurodegeneration, we analyzed the expression of pro-survival and pro-apoptotic proteins in a new group of animals that were sacrificed 24 hours after the last injection. As shown in Figure 5A, the expression of the pro-survival proteins Bcl-2 and Bcl-XL tended to increase in all the experimental conditions analyzed, being significantly higher in saline + LPS and LPS + LT rats (*P* <0.05; see Figure 5C). However, the expression of the pro-apoptotic proteins Bax and Bak (Figure 5B), was strongly and significantly increased exclusively in the LPS + LT animals (*P* <0.05; Figure 5C). On average, the pro-apoptotic to pro-survival ratio was six-fold higher in this group of animals compared to animals injected with saline + LPS or saline + LT, indicating that LPS-induced neuroinflammation increases the susceptibility to cellular death after proteasome inhibition. In fact, Fluoro-Jade B staining revealed that the saline + LPS injection produced minimal neurodegeneration, just in some neurons surrounding the track of injection, whereas the saline + LT injection induced a moderate neurodegeneration, mostly in the pyramidal cells of the CA3 region (Figure 6A; see also [22]). However, and in agreement with biochemical data, the LPS + LT injection strongly increased the number of Fluoro-Jade-B-positive cells throughout the hippocampus. Importantly, most of the degenerative signal was observed in the dentate gyrus, whereas in the CA3 region the number of Fluoro-Jade-B-positive cells was scarce, probably because of the higher susceptibility of this hippocampal region to proteasome inhibition, as we have previously shown to occur in aged rats [22]. Indeed, cresyl violet staining revealed a patent decrease in the number of pyramidal cells in both the CA1 and CA3 regions of the LPS + LT rats (Figure 6A, panel c), and most clearly, the expression of the neuronal marker NeuN was practically absent in the LPS + LT-injected rats, compared to both the saline + LPS and the saline + LT animals (Figure 6A, panels d-f). Finally, the neurodegenerative effect produced by the combination of neuroinflammation and proteasome inhibition could be mediated, at least in part, by apoptosis, as revealed the higher processing of caspase 3 in the LT + LPS animals (Figure 6B).

## Discussion

In the present work we have evaluated the potential role of neuroinflammation as a synergic risk factor for hippocampal neurodegeneration induced by proteasome inhibition. Our results demonstrated that LPS injection, in addition to the classical neuroinflammatory response characterized by the production of proinflammatory mediators, also altered protein homeostasis. In fact, LPS injection produced neuronal accumulation of ubiquitinated proteins; *de novo* i-proteasome biogenesis; a transient decrease of the proteasome activity; and a robust and sustained transcriptional up-regulation of the E2 ubiquitin-conjugating enzyme UB2L6. Thus, the LPS-induced accumulation of ubiquitinated proteins in hippocampal neurons could be consequence of both increased ubiquitin conjugation activity, to meet the substrate demands of a strongly up-regulated antigen presentation machinery, and the shift from constitutive to i-proteasome upon neuroinflammation, to increase the peptide supply for antigen presentation [21,23,26-29]. Taken together, these data indicate that LPS-induced modification of protein homeostasis could be part of a more general neuroinflammatory response to increase the production of peptides for antigen presentation [17,30]. In support of this, viability of neurons was well-preserved during the adaptation to this short phase of reduced proteolytic activity, as judge by the absence of Fluoro-Jade B staining and the predominant expression of pro-survival proteins (see also [2]). However, under this scenario (neuroinflammatory response), neurons become more vulnerable to proteasome inhibition. Indeed, proteasome inhibition after neuroinflammation (LPS + LT) leads to an early and predominant expression of pro-apoptotic proteins, followed by a qualitative increase in the number of degenerating neurons. Moreover, this combined treatment produced the formation of aggresome-like structures exclusively in the LPS + LT-treated rats. Similar structures have been previously observed *in vitro* after treatment with IFN-γ in cells lacking i-proteasome, suggesting that i-proteasome plays a pivotal role in both cytokine-mediated inflammation and the clearance of damage proteins and aggresome-like structures [24]. In this sense, our *in vivo* results are supporting this view. LPS injection produced a sustained expression of i-proteasome and the accumulation of ubiquitinated proteins in hippocampal neurons without the appearance of aggresome-like structures. However, proteasome inhibition in rats expressing a higher proportion of i-proteasome (that is, following LPS injection) led to the formation of neuronal aggresome-like structures. Importantly, all these biochemical and cellular modifications were not observed when proteasome inhibition or neuroinflammation were induced separately. Thus, present data strongly indicate that neuroinflammation is acting as a synergic risk factor for intracellular protein accumulation and neurodegeneration.

Having in mind that acute neuroinflammation induced by LPS injection in young animals is not at all similar to neuroinflammatory processes occurring during normal aging (chronic neuroinflammation), present findings could be relevant in the context of hippocampal aging. In this sense, aged rat hippocampus is characterized by chronic neuroinflammation [2,4,31]; decreased proteasome activity [32,33]; accumulation of ubiquitinated proteins [20,22]; higher proportion of i-proteasome [21]; and absence of significant neurodegeneration of pyramidal neurons [2]. Interestingly, LT injection alone in aged hippocampus reproduced protein accumulation observed in young rats injected with LPS + LT. Moreover, as we have previously shown, LT injection in aged rats leads to a predominant expression of pro-apoptotic proteins Bax, Bak and caspase-3 in addition to a higher neurodegeneration compared to young rats subjected to LT injection [22].

All of these age-related modifications can be reproduced in young animals only when proteasome was inhibited during the development of a neuroinflammatory response (LPS + LT). Thus, present and previous data support the idea that chronic neuroinflammation, as occurs in normal aging and in age-related neurodegenerative diseases (see below), should be considered as a synergic risk factor for neurodegeneration under situations of proteasome dysfunction (see [34] for a detailed review). In consequence, the modulation of neuroinflammation could represent an attractive therapeutic target in order to delay the onset and/or progression of neurodegeneration associated to the age-related neurodegenerative diseases [34]. Neuronal i-proteasome expression, which is almost absent in young healthy human brains, has been detected in the hippocampus from elderly patients as well as in patients affected by Alzheimer’s disease [35]. i-proteasome expression has also been observed in neurons localized in different brain areas from patients with Huntington’s disease, multiple sclerosis and temporal lobe epilepsy, diseases coursing with chronic neuroinflammation [36-38]. This raises the question whether i-proteasome expression has a protective role, as a homeostatic attempt of neurons to cope with the progressive accumulation of damage proteins, or, by contrast, has a deleterious effect. In this sense, present and previous data show evidence for both possibilities: i-proteasome expression may have a protective role in the context of acute neuroinflammation, but a detrimental effect when neuroinflammation become chronic as occurs in the majority of neurodegenerative diseases.

## Conclusions

The mechanisms underlying the neuroprotective role of i-proteasome in the context of acute neuroinflammation and its detrimental properties in the context of chronic neuroinflammation are currently unknown. The comprehension of the physiological role of i-proteasome in neuroinflammation and its participation in neurodegenerative diseases coursing with neuroinflammation is an expanding area in biomedical research. In this sense, the combination of neuroinflammation and proteasome inhibition may be a plausible model for the study of the physiological role of i-proteasome upon neuroinflammation and their involvement in diseases with a neuroinflammatory component. In summary, we report evidence supporting the idea that the two main hallmarks of age-related neurodegenerative diseases may form a neurodegenerative loop.

## Competing interests

The authors declare that they have no competing interests.

## Authors’ contributions

CP, EG and LG-C performed the research; MPG and AC designed and performed the research and analyzed the data. AG, JV and RMR designed the research; DR designed and supervised the research, analyzed the data and wrote the paper. All authors have read and approved the final version of the manuscript.
